# PHL8/445: Internet Courses for Cancer Patients

**DOI:** 10.2196/jmir.1.suppl1.e89

**Published:** 1999-09-19

**Authors:** A Forbriger

**Affiliations:** ^1^INKA- Cancer Information Network for Patients and their Families, HamburgGermany

**Keywords:** Cancer, Courses, Education, Patients, Public Adult Education

## Abstract

In September 1998 INKA started the first practical courses on the internet for cancer patients and their families in Germany. The aim was to show how they can use the internet to take more active control over the healing process. The project was open to all cancer patients and situated at the VHS Hamburg West (public adult education institute). Thanks to our sponsor the course fee was subsidised.85 participants took part. Most of them were cancer patients, with varying types of cancer. 2/3 were female and attended the course together with a family member or friend. They were aged 25-76 years and some of them even travelled from afar. 1/2 of the patients had a poor prognosis and a rare type of cancer. Asked why they took part they answered that they were either just curious, could not accept their poor prognosis, wanted to do something actively, thought INKA was a great project they wanted to join or were sent by their doctors to find out more information. Most of them were complete beginners with no internet experience, some without any computer knowledge. Apart from the technical introduction into the internet, its terms, retrieval methods etc. the participants had also enough opportunities for personal exchange within the group. Some keep in contact with each other and the project even now and 1/3 have their own internet access in the meantime. The patients and their families said that they were mostly dissatisfied with their interaction with their doctors and felt that the course was personally beneficial. The internet opened up a new dimension for them in the communication process, in that they learned that there is more and better information which they can print out and show to their doctors. They also liked to interact with experienced cancer survivors in the net. In general the course helped them to feel secure in their own healing decisions. Patients and institutions from all over Germany, Swiss and Austria showed interest and the conclusion is simple: there is a huge demand for this type of service. The project received a lot of press attention so that there were many multipliers in the health sector who now ask us for education material on this subject which INKA willing to provide in the future.

